# Case report: Two rare uterine cesarean scar mass cases

**DOI:** 10.1097/MD.0000000000033015

**Published:** 2023-03-24

**Authors:** Xiumin Zhao, Danjiang Huang, Dewen Yan, Xingxing Dai, Liping Wang

**Affiliations:** a Department of Gynaecology, Hangzhou Women’s Hospital, Hangzhou city, China; b Department of Radiology, Taizhou First People’s Hospital, Taizhou, China; c Department of Gynaecology, Taizhou First People’s Hospital, Taizhou, China; d Yuanqiao Central Health Center, Huangyan District, Taizhou City, China.

**Keywords:** case report, cesarean section scar pregnancy, choriocarcinoma, gestational trophoblastic neoplasia, magnetic resonance imaging

## Abstract

Gestational trophoblastic neoplasia (GTN) located in the cesarean scar is a rare disease that has imaging appearances similar to those of an exogenous scar incision pregnancy and is often misdiagnosed due to insufficient clinical experience.

**Patient concerns:**

We report 2 cases of uterine cesarean scar mass. Two patients with different diagnoses had similar clinical complaints as abnormal vaginal bleeding, enlargement of uterus isthmus by physical examination, and mixed echo mass in uterine low segment by ultrasound examination; however, their magnetic resonance imaging images showed very different features.

**Diagnoses:**

One patient was diagnosed with cesarean scar pregnancy (CSP) and one patient was diagnosed with cesarean scar GTN.

**Interventions:**

The CSP patient underwent surgery by laparoscopy combined with hysteroscopy after uterine artery embolism and obtained pathological confirmation. The GTN patient received chemotherapy.

**Outcomes:**

For the CSP patient, her serum β-human chorionic gonadotropin (hCG) concentration returned to normal 2 weeks later, and B-ultrasound showed that the niche was completely repaired 3 months after the operation. The intrauterine lesions of the GTN patient disappeared completely 3 months after serum β-hCG normalization. And her β-hCG was normal at all follow-up visits until now.

**Lessons:**

Clinicians should consider GTN when identifying masses at scar incision sites. Magnetic resonance imaging images improve the understanding of the imaging features in patients suspected of having CSP/GTN.

## 1. Introduction

Gestational trophoblastic neoplasia (GTN) is the only solid tumor that does not require pathological confirmation. It is also a highly chemosensitive tumor type that has a very good prognosis, even in the advanced stages. Due to the increase in cesarean rates worldwide, the incidence of cesarean section scar pregnancy (CSP) has increased gradually. In 2014, Qian et al reported the first case of cesarean scar choriocarcinoma that was initially misdiagnosed as CSP.^[1]^ This disease is an extremely rare entity, as a gestational trophoblastic disease in the cesarean scar is reported to have an incidence of only 1.5 per million pregnancies.^[2]^ Due to this very low incidence, few cesarean scar GTN patients can obtain a clinical diagnosis before pathological examination, especially GTNs following nonmolar pregnancy, and this delay often leads to heavy bleeding or hysterectomy.^[1,3]^ Here, we share our experience in the diagnosis and treatment of 2 patients with uterine cesarean scar mass: the first patient was diagnosed with CSP, and the second was diagnosed with cesarean scar GTN. Medical history, beta-human chorionic gonadotropin (hCG) measurements, and magnetic resonance imaging (MRI) helped us make the correct preoperative diagnosis.

## 2. Case report

**Figure 1.**
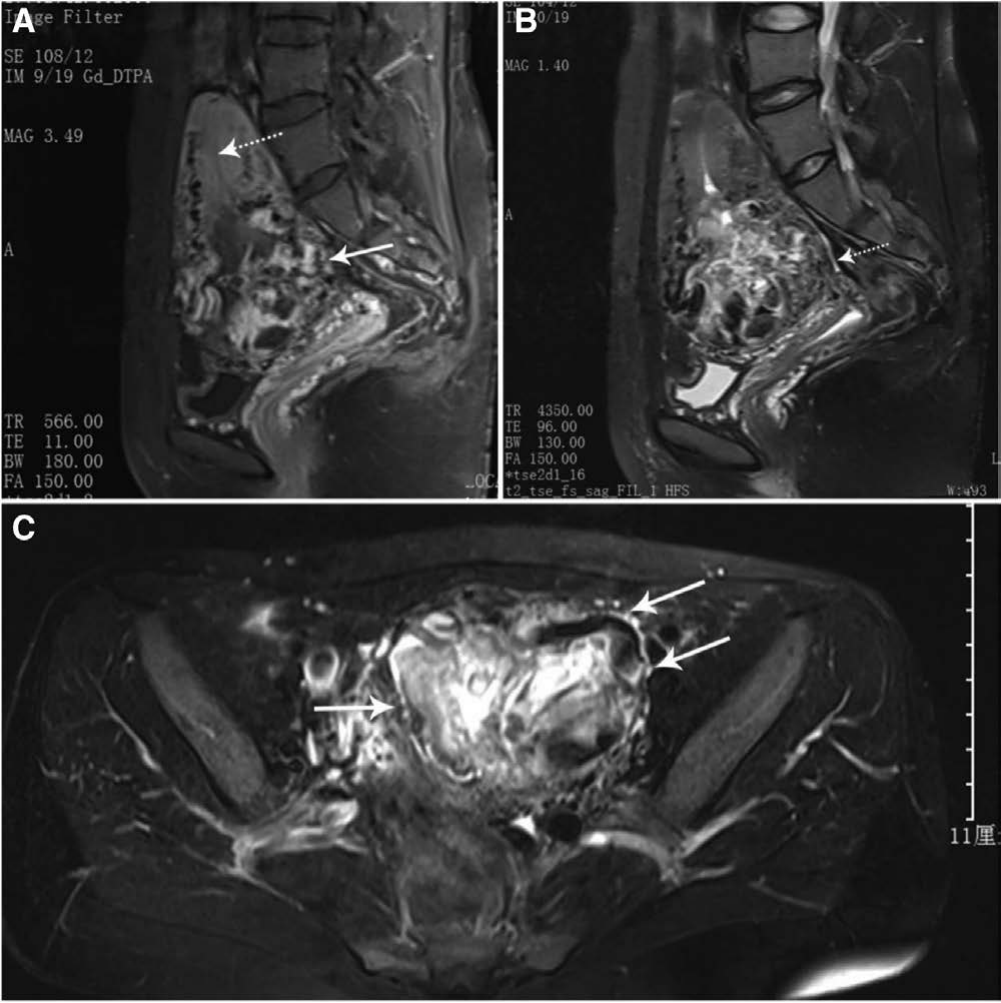
MRI images for the CSP patient. (A) Sagittal T1-weighted MRI showing a uterine mass (solid arrow) at the C-section scar with an empty uterine cavity (dashed arrow). (B) Sagittal T2-weighted MRI showing that the mass has some complex echo spaces that were due to hemorrhage or necrotic tissue, cysts, or vascular spaces (dashed arrow). (C) Coronal T2-weighted MRI showing the mass with abundant blood flow signals (solid arrow). CSP = cesarean section scar pregnancy, MRI = magnetic resonance imaging.

Case 1: A 36-year-old female patient, gravida 2, para 1, abortus 1, was admitted to our department in June 2017. She had delivered her first baby by cesarean section 6 years ago. In the 6th week of her current pregnancy, a B-ultrasound examination at the local hospital showed a gestational sac-like structure in the lower part of the uterus without fetal cardiac activity. The patient came to our hospital complaining of mild uterine hemorrhage lasting for 2 days presented at 8 weeks of gestation. She underwent transvaginal ultrasonography first which showed a mixed echo mass with a rich blood supply in the lower segment of the anterior uterine wall. Bimanual examination revealed a mildly enlarged uterus, the isthmus of the uterus was significantly enlarged, and the cervix was closed. The serum β-hCG concentration was 603 mIU/mL. MRI showed hemorrhage and tissue necrosis in the mass, which was surrounded by peritrophoblastic vasculature as the blood flow signal was abundant (Fig. 1a,b,c). CSP was first considered and GTD was ruled out by pathological diagnosis. The patient underwent pregnancy lesion resection and cesarean scar repair surgery by laparoscopy combined with hysteroscopy after uterine artery embolism. The intraoperative bleeding volume was 1500 mL, and the pathological diagnosis was pregnancy tissue. The serum β-hCG concentration returned to normal 2 weeks later, and B-ultrasound showed that the niche was completely repaired 3 months after the operation.

**Figure 2.**
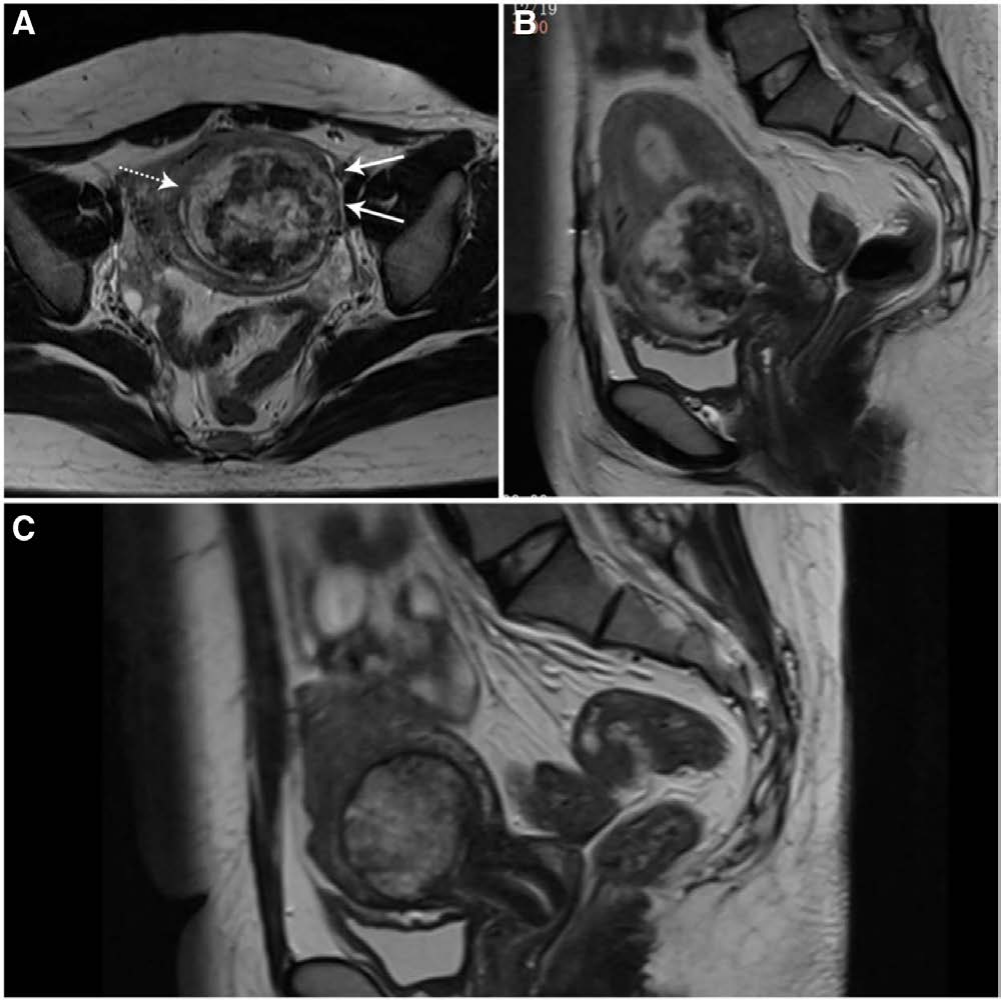
MRI images for the choriocarcinoma patient. (A) Coronal T2-weighted MRI showing the mass at the C-section scar with abundant blood flow signals around the lesion (solid arrow) and well-defined tumor margins (dashed arrow). (B) Sagittal T2-weighted MRI showed a mass without intratumoral vascularity due to severe central necrosis and hemorrhage. (C) Sagittal T2-weighted MRI images showed a gradual reduction of the solid uterine mass after 2 months of chemotherapy. MRI = magnetic resonance imaging.

Case 2: A 25-year-old female, gravid 4, para 2, abortus 1, with 2 previous cesarean section deliveries, was admitted to our department in April 2021. The patient was referred to the local hospital on 2 February 2021 because of persistent mild uterine hemorrhage lasting for 20 + days in her 10th week of pregnancy. Her hCG was 86698.13 mIU/mL, and B-ultrasound showed a 6.0 × 2.5 cm mass with mixed echoes in the uterus. She was diagnosed as having a miscarriage and was given mifepristone 150 mg + misoprostol 600 mg for abortion. On February 5, 2021, hysteroscopic curettage was performed to confirm that the lesion was completely removed, but pathological examination was not performed for the tissue collected by suction and curettage. Bleeding stopped 1 week after surgery; the patient underwent an hCG test once a week after surgery, with an hCG level of 800 mIU/mL on February 26, 2021. After that, the hCG level was not measured again. On March 19, 2021, she had mild vaginal bleeding but was not concerned as she thought it was her menses. On April 9, the patient was referred to our hospital due to complaints of consistent bleeding for 20 days. Ultrasonography showed a heterogeneous solid mass (6.5 × 5.6 × 5.9 cm) at the region of the patient’s cesarean section scar that was indistinguishable from the uterine myometrium, and a moderately rich blood flow spectrum was observed around the mass. Bimanual examination revealed a mildly enlarged uterus, marked enlargement of the left isthmus of the uterus, and closure of the cervix. The serum β-hCG concentration was 164501.66 mIU/mL, which increased to 195671.8 mIU/mL 3 days later. MRI showed a mixed mass in the lower uterus, and the periphery of the lesion was significantly enhanced under the contrast agent (Fig 2a,b). The patient’s thyroid function was normal. Computed tomography (CT) of the chest revealed several small nodules, the largest of which had a diameter of 7 mm; metastasis was highly suspected. The disease was staged and scored based on the International Federation of Gynaecology Obstetrics 2000 risk factor scoring/staging system for GTN (III: 7).^[4]^ The patient was given etoposide, methotrexate, dactinomycin/cyclophosphamide, and vincristine chemotherapy regimen. There was an exponential decrease in β-hCG levels from 195671.87 mIU/mL to 52602.19 mIU/mL after the first chemotherapy cycle. Serum hCG decreased to normal after 5 courses, the nodules on chest CT disappeared, and a progressive reduction in the lesion size was observed at the same time (Fig 2c). The patient received 1 course of etoposide, methotrexate, dactinomycin/cyclophosphamide, and vincristine and 2 courses of methotrexate for consolidation treatment. Three months after normalization, the intrauterine lesions disappeared completely. No obvious adverse reaction was found during chemotherapy. Her β-hCG was normal at all follow-up visits until now.

## 3. Discussion

GTN at the cesarean section scar is rare, which makes its diagnosis difficult. Wang et al retrospectively analyzed 938 GTN cases; only 13 (3.3%) cases were GTN located in the cesarean scar.^[5]^ These patients can be misdiagnosed as CSP, incomplete abortion, incomplete curettage, and placental remnants.^[6]^ In Case 2, the patient’s hCG level dropped to 800 mIU/mL after hysteroscopic curettage and then increased rapidly to 164501 mIU/mL 40 days later, which was highly suggestive of GTN.

Both patients underwent ultrasound as the first examination. Their ultrasounds were similar: a mixed-echo mass was observed at the cesarean section scar, which was indistinct from the myometrium of the uterine cavity and had a rich peripheral blood supply. GTN was not considered. Ultrasound is usually the initial diagnostic imaging test for CSP and GTN because of its convenience and repeatability. However, we think the primary diagnosis of GTN in a cesarean scar by ultrasound is very difficult. Qian et al^[1]^ reported the first case of cesarean scar choriocarcinoma had been misdiagnosed by ultrasound as CSP. Zhou et al^[7]^ reported 2 cases: a case of cesarean scar GTN and a case of CSP; both cases were misdiagnosed by ultrasound at first, but MRI led to the correct diagnosis. Gromi^[8]^ made the GTD diagnosis by ultrasound at the first examination because the mass showed the typical snowstorm sign.

MRI imaging has been recommended as a promising tool for noninvasive morphological analysis for CSP and for differentiating invasive mole from choriocarcinoma.^[9]^ The completely different MRI images of the 2 patients provided an important basis for our diagnosis. In Case 1, the MRI image showed substantial scattered anechoic lacunae in the chorion, named blood sinuses, the chorionic villi were degenerated and formed hydropic villi, appearing as vesicles or part of a heterogeneous mass. In Case 2, the MRI image showed nodular and well-defined tumor margins, absent or minimal intratumoral vascularity due to severe central necrosis and hemorrhage, and no tiny cystic lesions in or around the mass; these features were similar to those of other choriocarcinoma cases.^[5,9,10]^ Because choriocarcinoma usually invades the myometrium through the venous sinus, the tumor margin is nodular, and the boundary is usually clear, which is more obvious after chemotherapy. At the same time, an invasive mole often shows a poorly defined infiltrating mass with tiny cysts as the molar tissue always directly invades the myometrium and blood vessels. We did not find obvious differences in the ultrasound diagnosis of our 2 patients, which differed from the MRI conclusions. This may be related to poor clinical experience for disease and limited ultrasound images for retrospective observation. Real-time dynamic scanning and more clinical experience will be helpful for diagnosis.^[11]^

The lungs are the most likely site of metastasis in GTN patients with a probability of 80%.^[4]^ The chest CT image of our GTN patient showed several tiny rounded pulmonary nodules that had clear boundaries and a uniform density, which were considered indicative of distant metastasis.^[10,12]^ Our patient’s pulmonary nodules disappeared after 5 courses of chemotherapy, which confirmed the diagnosis.

There was no pathology diagnosis for the patient’s tissue collected by hysteroscopic curettage, which led to difficulties in our diagnosis and staging. The rate of pathological diagnosis after abortion is low for a country with a large population like China where pathologists are limited. Some medical centers recommended doing curettage or hysteroscopy after UAE for patients suspected of GTN to obtain a histopathological diagnosis. However, surgery was highly dangerous as the lesion had extremely abundant vascularization and was mainly bulging toward the serosa, with almost no overlying myometrium layer. The patient in Case 1 showed heavy bleeding during the operation even after UAE and preventive ligation of the uterine artery. There was also a report that a patient who was misdiagnosed as CSP received laparoscopic hysterectomy at the initial treatment. However, the patient had a temporary recurrence and died of tumor progression 39 months later after the surgery, as the final diagnosis was GTN in the caseation scar. For the patient in Case 2, we believed chemotherapy was acceptable to preserve her fertility as Tambe et al had done,^[3]^ and histopathological diagnosis is not mandatory for GTN patients.

In summary, early diagnosis of GTN in a cesarean scar is difficult because of its low incidence and nonspecific symptoms. Medical history, dynamic β- hCG monitoring, imaging examination, especially MRI, and metastasis assessment may be helpful in differential diagnosis. Making a correct clinical diagnosis by imaging but not pathology is undoubtedly useful for preserving patient fertility. We believe our experience in the diagnosis and treatment of patients with masses at the scar incision is helpful for the early diagnosis of scar incision GTN. Pathological diagnosis is recommended for every patient undergoing curettage or abortion.

## 4. Conclusion

Clinicians should consider GTN when identifying masses at scar incision sites. MRI is a useful tool for the diagnosis of CSP and GTN.

## Acknowledgments

Thanks to Dr Yanni Xiang and Dr Jie Lin of the Ultrasound Department of Taizhou First People’s Hospital for their assistance in the diagnosis of these diseases.

## Author contributions

**Data curation:** Danjiang Huang, Dewen Yan, Xingxing Dai.

**Supervision:** Liping Wang.

**Writing – original draft:** Xiumin Zhao.
